# HTLV-1 and Pregnancy: A Retrospective Study of Maternal and Neonatal Health Outcomes in an Endemic Region of Brazil

**DOI:** 10.3390/pathogens14040389

**Published:** 2025-04-16

**Authors:** Jacielma de Oliveira Freire, Maria Aparecida Figueredo Rodrigues, Greice Carolina Santos da Silva, Hugo Saba Pereira Cardoso, Marcio Luis Valença Araújo, Aloísio Santos Nascimento Filho, Briena Rodrigues Santos, Maria da Conceição Chagas de Almeida, Bernardo Galvão-Castro, Maria Fernanda Rios Grassi

**Affiliations:** 1 Escola Bahiana de Medicina e Saúde Pública—EBMSP, Salvador 40290-000, Bahia, Brazil; jacielma.freire@ebserh.gov.br (J.d.O.F.); maria1.rodrigues40@gmail.com (M.A.F.R.); brienna01@hotmail.com (B.R.S.); bgalvao@bahiana.edu.br (B.G.-C.); 2 Maternidade Climério de Oliveira, Universidade Federal da Bahia-UFBa/Empresa Brasileira de Serviços Hospitalares (EBSERH), Salvador 40055-150, Bahia, Brazil; 3 Fundação Oswaldo Cruz, Instituto Gonçalo Moniz—Fiocruz/IGM, Salvador 40296-710, Bahia, Brazil; greice.carolina@fiocruz.br (G.C.S.d.S.); conceicao.almeida@fiocruz.br (M.d.C.C.d.A.); 4 Departamento de Ciências Exatas e da Terra, Universidade do Estado da Bahia—UNEB, Salvador 41150-000, Bahia, Brazil; hugosaba@gmail.com; 5 Serviço Nacional de Aprendizagem Industrial, Campus Integrado de Manufatura e Tecnologias—SENAI CIMATEC, Salvador 41650-010, Bahia, Brazil; aloisio.nascimento@gmail.com; 6 Instituto Federal da Bahia—IFBA, Salvador 40301-015, Bahia, Brazil; marcioaraujo@ifba.edu.br

**Keywords:** HTLV-1, vertical transmission, pregnancy outcomes, neonatal health, Brazil

## Abstract

Human T-cell lymphotropic virus type 1 (HTLV-1) infection poses significant challenges to maternal and neonatal health, particularly in endemic regions. Vertical transmission, which occurs most commonly through prolonged breastfeeding and rarely during pregnancy, or childbirth, perpetuates the virus within families. This observational, retrospective study analyzed HTLV-1-infected and uninfected pregnant women admitted for delivery at a university maternity hospital in Salvador, Brazil (2020–2022). Medical records provided sociodemographic, clinical, and laboratory data. The HTLV-1 infection rate was 4.61 per 1000 deliveries. The sociodemographic characteristics were similar between infected (*n* = 17) and uninfected (*n* = 34) women. HTLV-1-positive women had higher rates of unplanned and undesired pregnancies. Adverse pregnancy outcomes were frequent in both groups (94.1% vs. 91.2%), but metabolic disorders and hypertension/eclampsia were more common among the infected women. Preterm birth and postpartum complications were also more frequent (17.6% vs. 5.9%, respectively), although the difference was not statistically significant. Breastfeeding initiation within the first hours of life was lower among exposed newborns (28.6% vs. 70%; *p* = 0.013). Neonatal characteristics did not differ significantly between the groups. These findings highlight critical gaps in reproductive health awareness and barriers to accessing preventive interventions. Further research on therapeutic strategies is urgently needed to support the World Health Organization’s (WHO) goal of eliminating HTLV-1 vertical transmission by 2030.

## 1. Introduction

Human T-cell lymphotropic virus type 1 (HTLV-1) is associated with a range of debilitating conditions, including adult T-cell leukemia/lymphoma (ATLL) [1], HTLV-1-associated myelopathy/tropical spastic paraparesis (HAM/TSP) [2,3], uveitis [4], and other inflammatory conditions, such as bronchiectasis and thyroiditis [5]. HTLV-1 infection also increases susceptibility to other infections, such as tuberculosis [6] and disseminated strongyloidiasis [7], contributing to higher morbidity and mortality among affected individuals [8].

HTLV-1 is transmitted through contaminated blood, and unprotected sexual intercourse, particularly from men to women, and from mother to child [9]. Vertical transmission occurs primarily through prolonged breastfeeding and plays a crucial role in the spread and persistence of the virus within families in endemic areas [10]. The risk of vertical transmission is associated with several factors, including high HTLV-1 proviral load in the mother’s blood or breast milk, elevated anti-HTLV-1 antibody levels, major histocompatibility complex compatibility, the presence of HAM/TSP, co-infection with *Strongyloides stercoralis*, and lower income [11]. Vertical transmission has also been linked to the development of ATLL in 1–5% of infected children, as well as to infectious dermatitis and the juvenile form of HAM/TSP [12,13]. Moreover, pregnancy may influence disease progression in women infected with HTLV-1, potentially favoring the development of HTLV-1-associated conditions [14].

The prevalence of HTLV-1 among pregnant women varies significantly between geographical regions. Endemic areas such as Japan show a prevalence of around 0.1% [15,16]. In Central and South America, including the Caribbean, the prevalence rate is 1.30%, while in specific regions of the Peruvian Amazon, a rate of 1.7% has been reported [17]. Large variation is found in Africa, with rates of 3.3 to 7.7% in Nigeria, 2.1 to 6.8% in Gabon, and 0% in Tunisia and Eritrea [18]. In Iran, although the prevalence is comparatively lower, it remains notable, with an infection rate of approximately 0.2% [19]. Similarly, lower prevalence rates have been reported, ranging from 0.04% to 0.06%, in Greece and Spain [20,21,22]. Brazil is recognized as an endemic region for HTLV-1, with an estimated 800,000 to 2.5 million individuals affected by the virus [23]. In Brazil, the prevalence of HTLV-1 among pregnant women ranges from 0.1% to 1.05% [10], with the highest burden observed in the Northeast region (0.60%; 95% CI 0.37–0.97) [24]. In response to this public health concern, the state of Bahia, located in this region, has implemented systematic prenatal screening for HTLV-1 since 2011 [25].

Few studies have examined pregnancy outcomes among women infected with HTLV-1 and the results have been inconclusive. Rates of miscarriage and abortion among infected women show considerable variability. One study conducted in Bahia found no significant impact of HTLV-1 infection on pregnancy outcomes, reporting similar rates of miscarriage and abortion compared to uninfected women [26]. Another study reported a prior abortion rate of 26.8% among infected women, with 31.7% experiencing more than two abortions, although direct comparisons with uninfected populations were not provided [27]. Adverse pregnancy outcomes, including fetal and neonatal death, have been documented [28].

Despite the clinical and epidemiological significance of HTLV-1, particularly in endemic regions, its impact on pregnancy outcomes and neonatal health remains unclear. This study aimed to characterize HTLV-1 infection in a cohort of pregnant women and their newborns in an endemic region of Brazil and evaluate associated pregnancy outcomes and neonatal health indicators.

## 2. Materials and Methods

### 2.1. Area and Hospital Characterization

This study was conducted on pregnant women at the Climério de Oliveira University Maternity Hospital (Maternidade Cimério de Oliveira, MCO) of the Federal University of Bahia. Situated in Salvador, Northeastern Brazil, the MCO is the first university maternity hospital in the country. It serves as a referral center for high-risk pregnancies and has 79 beds, including 10 in the Neonatal Intensive Care Unit and 13 in the Intermediate Care Unit. As part of Brazil’s public healthcare system (SUS), the MCO performs approximately 1500 deliveries annually and provides care to pregnant and postpartum women as well as newborns in both outpatient and inpatient settings.

### 2.2. Study Design

This observational, retrospective study was conducted from 1 January 2020, to 31 December 2022. Cases were defined as pregnant women infected with HTLV-1, while controls comprised uninfected pregnant women. Cases and controls were not matched. To enhance statistical power in the analysis of the clinical and sociodemographic characteristics, a 1:2 case-to-control ratio was established. Controls were selected sequentially during each recruitment year until the number of controls reached twice that of the cases.

### 2.3. HTLV Diagnosis

Blood samples from pregnant women were collected during prenatal care. Screening for HTLV was conducted using ELISA/chemiluminescence methodologies. All cases with positive or indeterminate results were confirmed using Western blot. HTLV test results were retrieved from medical records.

### 2.4. Participants

All pregnant women admitted to the MCO facility underwent screening for HTLV-1 infection. Cases with confirmed infection were reported to Sinan (Sistema de Informação de Agravos de Notificação), a national database of mandatorily notifiable diseases. Pregnant women with confirmed HTLV-1 who were admitted to the maternity hospital for delivery were included in the HTLV-1-positive group. The HTLV-1-negative group consisted of uninfected pregnant women. Pregnant women with HTLV-1 infection who did not give birth at MCO, as well as those with indeterminate Western blot or without a confirmatory HTLV-1 result by Western blot, were excluded.

### 2.5. Data Collection

Clinical and laboratory data, along with pregnancy characteristics, were obtained through a comprehensive review of medical records. The sociodemographic variables of the pregnant women, as well as clinical variables for both the mothers and newborns, were collected and completed using pregnancy and delivery data available in the medical records, notification forms, and the obstetric center’s delivery logbook. Data collection encompassed the entire prenatal follow-up period until delivery.

### 2.6. Sample Size Calculations

The sample size calculation was conducted considering a population size of 1500 (deliveries/year), margin of error (E) of 5% (0.05), confidence level of 95% (corresponding to a z-value of 1.96), and an estimated proportion of HTLV infection in pregnant women in Bahia (*p*) of 1% [10]. Given the specified margin of error and confidence interval, a sample size of 16 pregnant women was sufficient to ensure data reliability in assessing the characteristics of HTLV infection in pregnant women.

### 2.7. Data Analysis

Data analysis was conducted using SPSS software (version 17.0) for Windows, with categorical variables presented as frequencies and percentages. The groups were defined according to HTLV status, categorizing pregnant women as HTLV-positive or HTLV-negative and neonates as exposed or unexposed. To compare the groups, the Pearson Chi-square test was used, and Fisher’s exact test was applied for frequencies less than 5, with a *p*-value ≤ 0.05 considered statistically significant. The qualitative variables were categorized, resulting in 15 dichotomous variables and 3 polytomous variables. Normality was tested for the quantitative variables. As the data did not follow a normal distribution, the Mann–Whitney U test for independent samples was used to compare the groups. The relative risk (RR) was calculated as a measure of association to compare the likelihood of outcomes between the two groups (HTLV-positive and uninfected pregnant women), as well as between the exposed and unexposed newborns. Graphs were generated using GraphPad Prism software (version 8.0).

## 3. Results

### 3.1. Sociodemographic Characteristics and HTLV-1 Infection Among Pregnant Women

Between 2020 and 2022, a total of 3690 deliveries were recorded at the MCO (701 in 2020, 1186 in 2021, and 1803 in 2022). Among them, 51 pregnant women met the inclusion criteria: 17 in the HTLV-1-positive group and 34 in the HTLV-1-negative group. The overall rate of HTLV-1 infection among pregnant women during this period was 4.61 per 1000 deliveries.

The sociodemographic characteristics of the two groups were similar (Table 1). However, the median age was slightly higher among HTLV-1-positive women (30 vs. 26 years), and a greater proportion were unemployed (64.7%), although these differences were not statistically significant. The majority of the participants in both groups were non-white, married or in a common-law relationship, born and residing in the capital of Bahia, and had 10–12 years of formal education. The number of previous pregnancies, deliveries, and abortions did not differ significantly between groups.

Regarding the timing of HTLV-1 infection diagnosis, 35.3% of infected women were diagnosed before pregnancy, 11.8% during prenatal care, 29.4% at the time of delivery, and 23.5% were not informed.

### 3.2. Characteristics and Outcomes of the Current Pregnancy

HTLV-1-positive women reported a significantly higher frequency of unplanned (88.2% vs. 48.5%; *p* = 0.012) and undesired pregnancies (41.2% vs. 15.2%; *p* = 0.047) compared to HTLV-1-negative women (Table 2). However, no significant differences were observed between the groups in terms of the number of prenatal visits, location of consultations, gestational complications, gestational age at delivery, type of delivery, or fetal presentation.

Postpartum complications were more frequent in the HTLV-1-positive group; however, the difference was not statistically significant. Adverse pregnancy outcomes were reported in 94.1% (16/17) of HTLV-1-positive and 91.2% (31/34) of HTLV-1-negative women.

HTLV-1-positive pregnant women exhibited a higher prevalence of metabolic disorders (43.7% vs. 32.3%) and hypertension/eclampsia (25% vs. 19.3%), although these differences were not statistically significant (*p* > 0.05). Asthma and intrauterine growth restriction were more common in the HTLV-1-positive group (18.7% vs. 9.7%).

**Figure 1:**
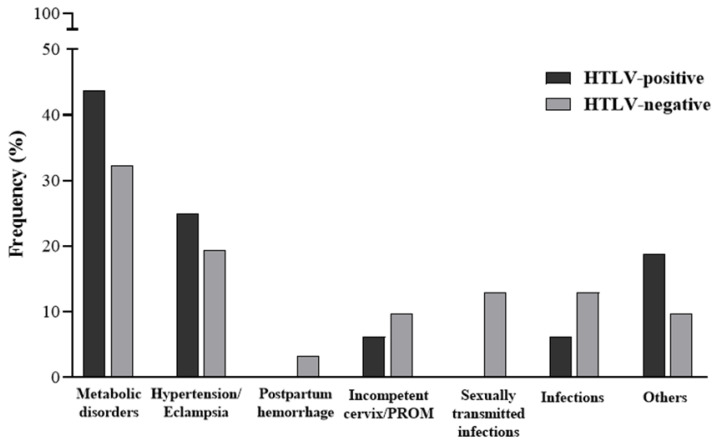
Adverse pregnancy outcomes of HTLV-1 infected (*n* = 16) and non-infected (*n* = 31) pregnant women. Metabolic disorders: obesity, gestational diabetes, and hyperthyroidism. Sexually transmitted infections: Hepatitis B, HIV, syphilis, and herpes. Infections: Chorioamnionitis, toxoplasmosis, and urinary tract infection. Other: asthma and intrauterine growth restriction. Chi-square: *p* > 0.05.

Conversely, HTLV-1-negative pregnant women had a higher frequency of postpartum hemorrhage (3.2% vs. 0%), cervical incompetence/premature rupture of membranes (9.7% vs. 6.2%), sexually transmitted infections (12.9% vs. 0%), and other infections (12.9% vs. 6.5%) (Figure 1). Although these conditions were more prevalent in the HTLV-1-negative group, the differences were not statistically significant.

### 3.3. Clinical Characteristics of Newborns Exposed and Not Exposed to HTLV-1

No significant differences were observed between HTLV-exposed and HTLV-unexposed newborns regarding gestational age, sex, birth weight, Apgar scores at 1 and 5 min, congenital malformations, resuscitation, positive pressure ventilation, or postnatal complications (Table 3). However, a significant difference was observed in breastfeeding initiation: 70% of HTLV-unexposed newborns were breastfed within the first hours of life, compared to only 28.6% in the HTLV-exposed group (*p* = 0.013).

Among the four newborns with postnatal complications in the HTLV-unexposed group, two died. In contrast, no neonatal deaths were recorded in the HTLV-exposed group. Although the proportion of preterm births was higher among HTLV-exposed newborns (17.6% vs. 5.9%), this difference was not statistically significant (*p* > 0.05).

Importantly, 23.5% (4/17) of HTLV-1-positive women had medical records indicating a lack of awareness regarding their right to receive infant formula as an alternative to breastfeeding. Additionally, many women reported feelings of sadness, which were associated with both their HTLV-1 diagnosis and the breastfeeding restrictions imposed by the infection.

## 4. Discussion

In this study, we assessed the pregnancy characteristics of women infected with HTLV-1 and the neonatal health of their newborns. The infected and uninfected groups showed similarities in sociodemographic and clinical characteristics. However, women infected with HTLV-1 exhibited a higher frequency of adverse pregnancy outcomes than their uninfected counterparts, although the difference was not statistically significant. The prevalence of metabolic disorders (11.5-fold), hypertension/eclampsia (5.6-fold), asthma, and intrauterine growth restriction (9-fold) was higher among HTLV-1-infected women compared to uninfected women. Conversely, infections (6.6-fold), sexually transmitted diseases during pregnancy (13-fold), cervical incompetence, and premature membrane rupture (3-fold) were more frequent among uninfected women.

Few studies have examined the impact of HTLV-1 infection on the course of pregnancy and its outcomes in pregnant women and exposed children. For example, a study conducted in Salvador, Brazil, with nearly 5000 pregnant women, found no significant impact of HTLV-1 infection on pregnancy outcomes, reporting similar rates of miscarriage and abortion among infected and uninfected women [26]. Another study reported a prior abortion rate of 26.8% among infected women, with 31.7% experiencing more than two abortions, although direct comparisons with uninfected populations were not provided [27]. In the present study, we did not observe differences in the number of reported abortions between the HTLV-1-infected and uninfected groups. However, low birth weight (<2.5 kg) was three times more common among HTLV-1-exposed infants compared to unexposed infants. Conversely, unlike the findings of Barmpas et al. (2019), who reported a higher frequency of premature membrane rupture among HTLV-1-infected women, we observed this outcome more frequently among uninfected women [28].

A notable aspect of this study was the impact of HTLV-1 infection on the psychosocial well-being of pregnant women. A higher proportion of infected women reported unintended pregnancies. Furthermore, we observed that nearly a quarter of the pregnant women diagnosed with HTLV-1 breastfed their children, highlighting a lack of awareness regarding the transmission routes of HTLV-1 and its associated diseases. The World Health Organization (WHO) and Pan American Health Organization (PAHO) recommend breastfeeding for up to 24 months due to its well-documented benefits for child health and psycho-affective bonding [29]. However, prolonged breastfeeding is a primary route of the vertical transmission of HTLV-1 in endemic areas [30]. Approximately 20% of children born to infected mothers acquire the infection, with higher transmission rates among those breastfed for more than six months, although transmission has also been reported in infants breastfed for shorter durations [31]. Additionally, a systematic review found that 5% of children born to mothers living with HTLV-1 who were not breastfed still acquired the infection [32]. A key challenge in assessing the impact of vertical transmission is the delayed confirmation of HTLV-1 infection in exposed children. Unlike other perinatally transmitted infections, HTLV-1 diagnosis in infants cannot be confirmed at birth due to the persistence of maternal antibodies. Definitive diagnosis relies on serological and molecular testing at 18 months of age, making early detection unfeasible within the scope of this study. This limitation underscores the need for the long-term follow-up of HTLV-1-exposed infants to accurately determine their infection status and evaluate the associated health outcomes.

A critical barrier to preventing HTLV-1 vertical transmission is limited awareness of maternal serological status or delayed diagnosis. HTLV-1 antenatal screening as a national infection control policy has proven to be cost-effective and has the potential to reduce HTLV-1-associated diseases in endemic countries such as Japan and Brazil [33,34]. Since 2011, mandatory HTLV testing for pregnant women has been implemented in Bahia state, alongside lactation suppression therapy at delivery and free formula milk for exposed infants [35]. Women living with HTLV-1 must be aware of their serostatus so they can make informed decisions regarding breastfeeding. Access to comprehensive care and evidence-based information is essential to enable these women to weigh the risks and benefits and make autonomous choices free from societal pressure [36,37]. Recently, Brazil expanded this policy nationally, aligning with WHO and PAHO recommendations to eliminate the vertical transmission of HTLV-1 by 2030 [25]. However, despite these initiatives, some pregnant women remain unaware of their entitlement to free formula. This lack of awareness is likely linked to late diagnosis, as more than half of the women in this study were diagnosed during prenatal care or at delivery. Limited access to healthcare services, particularly in smaller cities, may contribute to knowledge gaps regarding HTLV-1 and available preventive services. Late detection hinders the implementation of preventive strategies, thereby increasing the risk of transmission to newborns.

The delayed diagnosis of HTLV-1 infection may result from barriers to serological testing, including geographical distance, financial constraints, and low awareness among healthcare professionals. Moreover, the centralization of confirmatory testing (Western blotting) contributes to diagnostic delays, limiting opportunities for timely intervention. The development of rapid diagnostic tests for HTLV-1 could enhance early detection and improve follow-up care for pregnant women. In endemic regions, public health policies must be strengthened to provide individualized care for women of childbearing age, with a specific focus on HTLV-1 screening, the prevention of vertical transmission, and comprehensive clinical and psychosocial support. In Brazil, public health policies are being progressively implemented to incorporate key strategies, including universal prenatal screening, confirmatory testing for reactive cases, mandatory notification, the establishment of multidisciplinary reference centers, expanded testing for high-risk populations, and public awareness campaigns [38]. Additionally, healthcare professionals must receive adequate training to recognize the signs and symptoms of HTLV-1 infection and provide appropriate counseling and testing.

A limitation of this study is the relatively small sample size, which may have reduced statistical power to detect significant differences between groups. While data on infant infection status would have provided an additional dimension of analysis, this study addresses a critical gap by focusing on maternal health. Understanding pregnancy outcomes in women infected with HTLV-1 is essential for optimizing prenatal care, mitigating potential risks, and informing future research on strategies to prevent mother-to-child transmission. The findings lay the groundwork for larger, prospective studies that could follow mother–child pairs to assess neonatal outcomes over time. Additionally, as the study population was drawn from a referral maternity hospital specializing in high-risk pregnancies, the high proportion of women with gestational hypertension and diabetes may have introduced selection bias, given the known associations of these conditions with adverse pregnancy outcomes.

## 5. Conclusions

The HTLV-1-infected women in this study experienced higher frequencies of preterm births and adverse pregnancy outcomes than uninfected women, particularly with regard to metabolic disorders. However, these differences were not statistically significant. Additionally, we identified major gaps in their knowledge regarding reproductive rights and access to preventive interventions. Further research on novel therapeutic strategies to reduce vertical transmission and improve maternal and neonatal outcomes is urgently needed. These efforts are essential for achieving the WHO’s and PAHO’s goals of eliminating HTLV-1 vertical transmission by 2030.

## Figures and Tables

**Table 1:** Sociodemographic data of HTLV-positive and HTLV-negative pregnant women.

Characteristic	HTLV-Positive *n* = 17	HTLV-Negative *n* = 34	*p*-Value
Age years, median (IQR)	30 (24–36)	26 (21–33)	0.161 ^a^
Ethnicity			0.663 ^b^
White	2 (11.8)	4 (11.8)	
Non-white	15 (88.2)	30 (88.2)	
Marital status			0.831 ^b^
Single	5 (29.4)	11 (32.4)	
Married/Co-habiting	12 (70.6)	23 (67.6)	
Region of origin			0.357 ^b^
Capital	12 (70.6)	27 (79.4)	
Countryside	5 (29.4)	7 (20.6)	
Place of birth			1 ^d^
Capital	9 (52.9)	18 (52.9)	
Countryside	8 (47.1)	15 (44.1)	
Other Brazilian state	0 (0)	1 (2.9)	
Years of schooling			0.429 ^d^
0–9	2 (11.8)	10 (29.4)	
10–12	13 (76.5)	21 (61.8)	
13+	2 (11.8)	3 (8.8)	
Occupation			0.074 ^c^
Employed	6 (35.3)	21 (61.8)	
Unemployed *	11 (64.7)	13 (38.2)	
Pregnancies			
1–3	12(70.6)	28 (82.4)	0.269 ^b^
3+	5 (29.4)	6 (17.6)	
Previous deliveries			0.379 ^d^
0	6 (35.3)	12 (35.3)	
1–3	10 (58.8)	17 (50)	
4+	1 (5.9)	5 (14.7)	
Previous miscarriages			0.576 ^d^
0	9 (52.9)	22 (64.7)	
1–3	8 (47.1)	11 (32.4)	
4+	0	1 (2.9)	
Gestational age at delivery	39 (36.5–40.5)	39 (37.7–40)	0.641 ^c^
Timing HTLV diagnosis			
Previous pregnancy	6 (35.3)	NA	NA
During prenatal care	2 (11.8)	NA	NA
At delivery	5 (29.4)	NA	NA
Not informed	4 (23.5)	NA	NA

Data are presented as numbers (*n*) and percentages (%). Gestational age delivery is presented as median (IQR). IQR, interquartile range (25–75th percentile); NA, not applicable. * Unemployed, homemaker, or student. ^a^ Mann–Whitney U test; ^b^ Fisher’s exact test; ^c^ Chi-square test, ^d^ Fisher, Monte Carlo, *n* = 10.000. *p* < 0.05.

**Table 2:** Characteristics and outcomes of the current pregnancy in HTLV-positive and HTLV-negative women.

Characteristic	HTLV-Positive *n* = 17	HTLV-Negative *n* = 34	RR (95% CI)	*p*-Value
Antenatal appointments *				
Up to 5	3 (18.8)	5 (15.2)	1.29 (0.27–6.24)	0.750 ^a^
6 or more	13 (81.2)	28 (84.8)	1	
Location of antenatal appointments *				
Capital	12 (75.0)	27 (79.4)	1	
Countryside	4 (25.0)	7 (20.6)	1.29 (0.32–5.24)	0.383 ^a^
Unplanned pregnancy *				
Yes	15 (88.2)	16 (48.5)	7.97 (1.57–40.50)	0.012 ^a^
No	2 (11.8)	17 (51.5)	1	
Undesired pregnancy *				
Yes	7 (41.2)	5 (15.2)	3.92 (1.01–15.21)	0.047 ^b^
No	10 (58.8)	28 (84.8)	1	
Pregnancy complications				
Yes	10 (58.8)	19 (55.9)	1.13 (0.35–3.67)	0.842 ^b^
No	7 (41.2)	15 (44.1)	1	
Delivery type				
Vaginal	11 (64.7)	17 (50.0)	1.83 (0.55–6.09)	0.087 ^b^
Cesarean	6 (35.3)	17 (50.0)	1	
Fetal presentation				
Cephalic	16 (94.1)	33 (97.1)	1	
Breech or Footling	1 (5.9)	1 (2.9)	2.06 (0.12–35.14)	0.560 ^a^
Postpartum complications *				
Yes	3 (17.6)	2 (5.9)	3.43 (0.51–22.84)	0.199 ^a^
No	13 (82.4)	29 (94.1)	1	

Data are presented as numbers (*n*) and percentages. RR: Relative risk; CI: Confidence interval. ^a^ Fisher’s exact test; ^b^ Chi-square test; *p* < 0.05. * Missing data are presented in Supplementary Table S1.

**Table 3:** Clinical characteristics of newborns exposed and not exposed to HTLV-1 when born to infected and uninfected pregnant women.

Characteristic	HTLV-Exposed *n* = 17	HTLV-Unexposed *n* = 34	RR (95%CI)	*p*-Value
Gestational age				
Preterm (<37 weeks)	3 (17.6)	2 (5.9)	3.43 (0.56–22.84)	0.199
Term (37–42 weeks)	14 (82.4)	32 (94.1)	1	
Sex *				
Female	10 (62.5)	13 (40.6)	1	
Male	6 (37.5)	19 (59.4)	2.44 (0.71–8.36)	0.153
Birth weight *				
Underweight (<2.5 kg)	3 (18.8)	2 (5.9)	3.69 (0.55–24.73)	0.311
Normal/Overweight (≥2.5)	13 (81.2)	32 (94.1)	1	
Apgar score at 1st minute *				
No/Mild difficulty	15 (88.2)	30 (90.9)	1	
Moderate/Severe difficulty	2 (11.8)	3 (9.1)	1.33 (0.20–8.86)	0.560
Apgar score at 5th minute *				
No/Mild difficulty	17 (100)	31 (93.9)	1	
Moderate/Severe difficulty	-	2 (6.1)	0.94 (0.86–1.02)	0.431
Congenital malformation	-			
Yes	0	2 (5.9)	0.94 (0.86–1.02)	
No	16 (100)	32 (94.1)	1	0.458
Resuscitation *				
Yes	1 (6.2)	2 (5.9)	1.07 (0.09–12.71)	0.695
No	15 (93.8)	32 (94.1)	1	
Positive pressure ventilation *				
Yes	2 (12.5)	4 (11.8)	1.07 (0.18–6.56)	0.635
No	14 (87.5)	30 (88.2)	1	
Breastfeeding in the first hours of life *				
Yes	4 (28.6)	21 (70.0)	1	
No	10 (71.4)	9 (30)	5.83 (1.44–23.61)	0.013
Postnatal complications *				
Yes	4 (23.5)	3 (9.4)	2.97 (0.58–15.24)	0.178
No	13 (76.5)	29 (90.6)	1	

Data are presented as numbers (*n*) and percentages (%). RR: Relative risk; CI: Confidence interval. * Missing data are presented in Supplementary Table S2. Fisher’s exact test, *p* < 0.05.

## Data Availability

All relevant data are within this paper and its Supporting Information files.

## Supplementary Materials

The following supporting information can be downloaded at: https://www.mdpi.com/article/10.3390/pathogens14040389/s1, Table S1: Missing data in the characteristics and outcomes of current pregnancy in HTLV-positive and HTLV-negative women; Table S2: Missing and excluded data in the clinical characteristics of HTLV-exposed and non-exposed newborns.

## Abbreviations

The following abbreviations are used in this manuscript:

HTLV-1	Human T-cell lymphotropic virus type 1
ATLL	Adult T-cell leukemia/lymphoma
HAM/TSP	HTLV-1-associated myelopathy/tropical spastic paraparesis
MCO	Maternidade Cimério de Oliveira
WHO	World Health Organization
PAHO	Pan American Health Organization
