# Supplementary material for: HTLV-1 and Pregnancy: A Retrospective Study of Maternal and Neonatal Health Outcomes in an Endemic Region of Brazil
Source: Pathogens. 2025 Apr 16;14(4):389. doi: 10.3390/pathogens14040389 (PMC12030755; doi:10.3390/pathogens14040389)
Supplement: Supplementary file 1 [file pathogens-14-00389-s001.zip › pathogens-3500600-supplementary.pdf]

Supplementary Table S1. Missing data in the characteristics and outcomes of current pregnancy in HTLV-positive and HTLV-negative women.

Variable	Missing cases		
	n (%)	HTLV-positive	HTLV-negative
Antenatal appointments	2 (3.90)	1	1
Location of antenatal appointments	1 (1.96)	1	0
Unplanned pregnancy	1 (1.96)	-	1
Undesired pregnancy	1 (1.96)	-	1
Postpartum complications	4 (7.80)	1	3

Data represent the number and frequency of missing cases, considering all 51 participants.

Supplementary Table S2. Missing and excluded data in the clinical characteristics of HTLV-exposed and non-exposed newborns

Variable	Missing cases		
	***n* (%)**	HTLV-exposed	HTLV-unexposed
Sex	3 (5.88)	1	2*
Birth weight	1 (1.96)	1	-
Apgar score at 1st minute	1 (1.96)	-	1
Apgar score at 5th minute	1 (1.96)	-	1
Congenital malformation	1 (1.96)	1	-
Resuscitation	1 (1.96)	1	-
Positive pressure ventilation	1 (1.96)	1	-
Breastfeeding in the first hours of life	7 (13.72)	3	4
Postnatal complications	1 (1.96)	-	2

* One intersex case was excluded in the HTLV-unexposed group. Data represent the number and frequency of missing cases, considering all 51 participants.
